# Treatment of Neuroendocrine Neoplasms with Radiolabeled Peptides—Where Are We Now

**DOI:** 10.3390/cancers14030761

**Published:** 2022-02-01

**Authors:** Mitesh Naik, Adil Al-Nahhas, Sairah R. Khan

**Affiliations:** Department of Imaging, Charing Cross Hospital, Fulham Palace Road, London W6 8RF, UK; a.al-nahhas@nhs.net

**Keywords:** neuroendocrine, neoplasms, NET, NEN, radionuclide, PRRT, lutetium, yttrium, gallium

## Abstract

**Simple Summary:**

Neuroendocrine neoplasms (NENs) are a rare and diverse group of malignancies which are rising in incidence. Several treatments have been devised for unresectable or metastatic tumors, including peptide receptor radionuclide therapy (PRRT). PRRT specifically targets cells that express high levels of somatostatin receptors, such as well-or moderately differentiated NENs, to enable precise delivery. This article highlights the journey of PRRT from inception to the present day, where it is now integral in clinical practice guidelines worldwide. It also provides an overview of NENs and a history of somatostatin receptor imaging, which facilitates the selection of patients for PRRT. Practical considerations relating to appropriate use, treatment administration and side-effects are discussed, and perspectives on future directions to boost efficacy are detailed.

**Abstract:**

Peptide receptor radionuclide therapy (PRRT) has been one of the most successful and exciting examples of theranostics in nuclear medicine in recent decades and is now firmly embedded in many treatment algorithms for unresectable or metastatic neuroendocrine neoplasms (NENs) worldwide. It is widely considered to be an effective treatment for well- or moderately differentiated neoplasms, which express high levels of somatostatin receptors that can be selectively targeted. This review article outlines the scientific basis of PRRT in treatment of NENs and describes its discovery dating back to the early 1990s. Early treatments utilizing Indium-111, a γ-emitter, showed promise in reduction in tumor size and improvement in biochemistry, but were also met with high radiation doses and myelotoxic and nephrotoxic effects. Subsequently, stable conjugation of DOTA-peptides with β-emitting radionuclides, such as Yttrium-90 and Lutetium-177, served as a breakthrough for PRRT and studies highlighted their potential in eliciting progression-free survival and quality of life benefits. This article will also elaborate on the key trials which paved the way for its approval and will discuss therapeutic considerations, such as patient selection and administration technique, to optimize its use.

## 1. Introduction

Neuroendocrine neoplasms (NENs) are a diverse group of malignancies, derived from multipotent stem cells, which have migrated primarily from the endoderm to tissues throughout the body [1]. They are a heterogenous group of neoplasms with a wide range of clinical presentations, with some producing symptoms depending on their location and others remaining dormant for extended periods. It is the latter which are more difficult to diagnose and can be advanced at presentation. They account for approximately 0.5% of all newly diagnosed malignancies and are most commonly found in the gastrointestinal tract or pancreas [2]. Their incidence is rising, in part thought secondary to improvements in diagnostic tests. Indeed, they may be detected incidentally due to their often-insidious presentation [3]. The neoplasms can be described as functioning or non-functioning, depending on their ability to overproduce bioactive hormones such as serotonin, insulin, gastrin and glucagon. It is these hormones which may contribute to the clinical symptoms in presenting patients, such as the typical ‘carcinoid syndrome’ from excessive serotonin secretion and metabolism. Inherited syndromes, such as von Hippel-Lindau, neurofibromatosis type 1 and multiple endocrine neoplasia type 1, may increase the likelihood of developing NENs [4]. There have been multiple classification systems used in recent decades, predominantly dividing tumors in groups according to their location and then subdividing by morphological characteristics and hormone functionality to determine their overall behavior [5].

Neoplasms were originally classified as foregut, midgut or hindgut depending on their embryonic origin, with foregut neoplasms developing in the respiratory system, thymus and upper gastrointestinal tract; midgut neoplasms developing in small bowl, appendix and ascending colon; and hindgut neoplasms in the distal colon and rectum [6]. This was subsequently found too simplistic and smaller groups based on anatomical location were preferred. Following improvements in understanding of the underlying histology of the neoplasm, iterations of the WHO classification at the turn of the century also discriminated according to the level of cellular differentiation. The term ‘differentiation’ is used to describe how closely the neuroendocrine cells resembled their non-neoplastic counterparts based on morphology and expression of neuroendocrine markers such as chromogranin A and synaptophysin [7]. The level of differentiation is thought to relate to the overall aggressiveness of the tumor, or grade, but the rate of proliferation has also been found to be prognostically significant; this was first adopted by the European Neuroendocrine Tumor Society (ENETS) in 2006 [8]. The WHO classification system has also since been adapted to include proliferative rates either assessed as the number of mitoses per unit area of neoplasm, or as the percentage of cells labelled with Ki-67, a marker of proliferation [9]. The most recent classification system of gastrointestinal and pancreatic NEN were produced by the WHO in 2019 and 2017 respectively, with pertinent differences between this and the 2010 iteration shown in Table 1. The most recent classification has clarified previous semantic issues, using NEN as an all-encompassing term for both well- and poorly-differentiation tumors of neuroendocrine cells, whilst the term neuroendocrine tumor (NET) is reserved for well-differentiated neoplasm and neuroendocrine carcinoma (NEC) is defined as a poorly differentiated neoplasm. Another important change is the inclusion of a high-grade category for well differentiated NETs (defined as a mitotic rate >20 per 2 mm^2^ or Ki-67 >20%) which are distinct from poorly differentiated NECs. Staging is performed using formal TNM-based systems independently produced by the American Joint Committee on Cancer (most recently the 8th edition) [10] and the ENETS, which are separated by tumor location.

Neuroendocrine neoplastic cells have long been described to overexpress somatostatin receptors (SSTRs), a family of G-protein-coupled-receptors [11,12]. There are five subtypes of the receptor; SSTR2 and SSTR5 are most commonly expressed, particularly in gastroenteropancreatic (GEP) NENs [13]. This forms the basis of treatment with synthetic somatostatin analogues such as octreotide and lanreotide, which preferentially bind to SSTR2 with high affinity and have a longer half-life than somatostatin itself. In turn this has been found to provide symptomatic relief and stabilization of tumor growth, but regression is rare [13]. Possible mechanisms underpinning the antiproliferative property of somatostatin analogues include antagonism of local growth factor release and indirect anti-angiogenetic effects, alongside intrinsic inhibition of hormone secretion. Two seminal papers comparing somatostatin analogues with placebo were the PROMID [14] and CLARINET [15] studies, randomized controlled studies in 90 patients with metastatic midgut and 204 patients with enteropancreatic tumors respectively. Both studies demonstrated improved progression-free survival in patients treated with somatostatin analogues. Curative surgical resection is only an option in a subset of patients; therefore, somatostatin analogues are central in treatment pathways for patients with NENs.

## 2. Somatostatin Receptor Imaging

After the overexpression of somatostatin receptors was initially detected in pituitary tumor tissue [16] and then in surgical samples of NENs [17], localization techniques were developed which exploited this finding by using radiolabeled ligands which bound to the receptors. This was first described in 1989, with in vivo imaging of NENs expressing somatostatin receptors with ^123^I-labelled Tyr-3-ocreotide [18,19,20]. 

Relatively high hepatic and intestinal uptake hampered interpretation of scintigraphic images acquired using this compound and the labelling process was onerous, so this was soon followed by the development and use of ^111^In-DTPA (Indium-111 diethylenetriaminepentaacetic acid)-octreotide or ^111^In-pentreotide, also known as Octreoscan [21]. This was, for many years, the gold standard in the diagnostic workup of GEP NENs following a study in a cohort of over 1000 patients which demonstrated high sensitivities: over 90% for carcinoids and 60–90% for pancreatic NENs depending on tumor type or lesion size [22]. Developments in gamma camera apparatus and a widespread increase in availability of single-photon emission computed tomography (SPECT) contributed to its ongoing use but limited spatial resolution for the detection of small tumors or those adjacent to sites of physiological uptake (e.g., spleen and kidneys), and the fact that ^111^In-DTPA had a relatively limited receptor affinity profile to SSTR2 predominantly, meant that further advances were deemed necessary [23].

**Figure 1:**
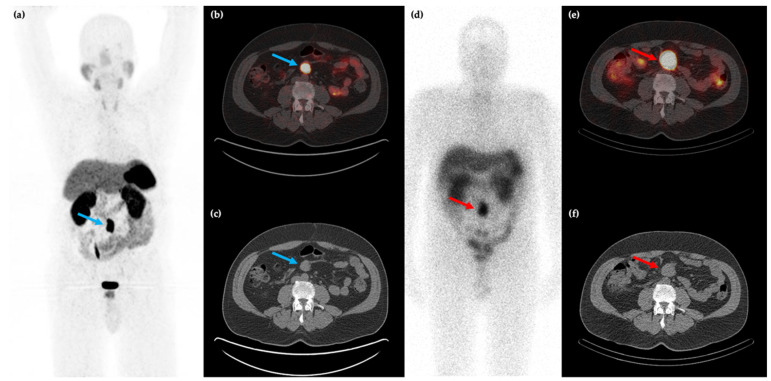
58-year-old male with a history of rectal bleeding and a mesenteric mass identified on conventional CT imaging. ^68^Ga-DOTATATE PET/CT maximum intensity projection (MIP) (**a**) and axial (**b**,**c**) images shows the somatostatin receptor (SSTR) positive lesion at the root of the small bowel mesentery (blue arrows) with improved spatial resolution compared to ^111^In-pentreotide SPECT/CT ((**d**–**f**), red arrows).

The application of the macrocyclic chelator DOTA (1,4,7,10-tetraazacyclododecane-1,4,7,10-tetraacetic acid) paved the way for use of positron emission tomography to image NENs, by combining it with positron emitter Gallium-68 (^68^Ga) and labelling this compound with somatostatin analogues to produce tracers collectively known as ^68^Ga-DOTA-peptides. ^68^Ga- DOTA-Tyr^3^-ocreotide (^68^Ga-DOTATOC) was the first tracer to show utility in imaging of NEN in 2001 [24], followed soon after by ^68^Ga-DOTA-Tyr^3^-octreotate (^68^Ga-DOTATATE) and ^68^Ga-DOTA-1-NaI^3^-octreotide (^68^Ga-DOTANOC) [25]. Positron emission tomography (PET) imaging using DOTA-conjugated somatostatin analogues posed several advantages, with higher spatial resolution and better image quantification with PET over SPECT (Figure 1) [26], and a higher affinity profile to SSTR of ^68^Ga-DOTA-peptides compared with ^111^In-DTPA-octreotide [27] which in turn improves detection of smaller lesions or those with lower SSTR expression [28]. Practical advantages include faster image acquisition, lower radiation dose [29], a longer half-life and better commercial availability of Germanium-68/Gallium-68 generators [30].

The affinity profile of the three major PET radiotracers for SSTR imaging varies; ^68^Ga-DOTATATE is most selective for SSTR2, ^68^Ga-DOTATOC binds with greater affinity to both SSTR2 and SSTR5 and ^68^Ga-DOTANOC with SSTR2, SSTR3 and SSTR5 [31]. Despite the differing affinity profiles there is no consensus opinion on the optimum tracer, and all are in clinical use. A meta-analysis described similar sensitivities of over 90% using both ^68^Ga-DOTATATE and ^68^Ga-DOTATOC [32]. These tracers have been particularly efficacious in assessment of G1-G2 GEP (gastro-entero-pancreatic) NENs due to high SSTR expression in almost 90%. Semi-quantitative image analysis using maximal standardized uptake value (SUV_max)_ has also shown potential to discriminate between G1-G2 and G3 NENs with lower SUV_max_ found in more aggressive tumors. A correlation has also been found between SUV_max_ and Ki-67 [33,34]. A reporting system for SSTR PET imaging, known as SSTR-RADS, has been proposed to standardize diagnosis and treatment planning, where uptake of lesions is compared to background liver and correlated on CT, giving a per-lesion score of 1 (benign) to 5 (NET almost certainly present) [35]. It has shown high interobserver agreement and adoption for trials and clinical use is thus supported [36], although this has not yet translated into practice.

**Figure 2:**
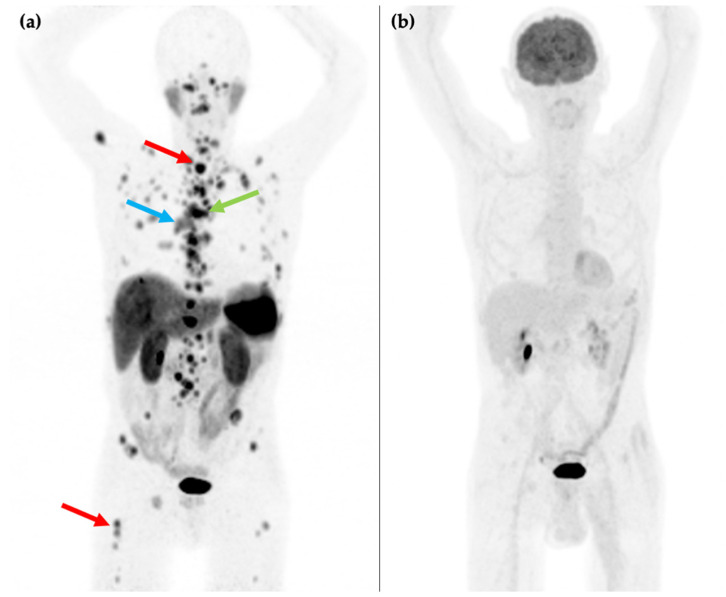
81-year-old male with an endobronchial lesion identified on conventional CT imaging. ^68^Ga-DOTATATE PET/CT MIP image (**a**) shows an SSTR positive right-sided bronchial lesion (blue arrow), mediastinal lymphadenopathy (green arrow), and innumerable bone metastases in the axial and proximal appendicular skeleton including in the spine and right femur (red arrows). ^18^F-FDG PET/CT MIP image (**b**) shows that the SSTR positive lesions are not FDG avid. Histology confirmed a G1 typical bronchial carcinoid (Ki-67 1–2%).

F-18 fluorodeoxyglucose (^18^F-FDG) PET has a role in imaging of high-grade NEN because of lower SSTR expression in these tumors [37,38]. As a glucose analogue, FDG uptake is proportional to metabolic activity and is associated with cellular proliferation. This corresponds to reduced survival and aggressive tumor behavior including a higher Ki-67 index [39,40]. It is thought to have little to no impact on treatment decisions for G1 NENs, as these exhibit no or minimal metabolic activity (Figure 2), and moderate impact on G2 NENs [41]. Consequently, its routine use is indicated in clinical guidelines [42] for high grade G2 and G3 tumors only [43,44]. It has been suggested that G2 tumors with a Ki-67 of over 10% should be imaged with ^18^F-FDG [45]. Although recent studies have assessed the value of combining ^18^F-FDG and ^68^Ga-DOTA-peptide PET/CT to stratify patients at major risk of progression, the heterogenous patterns of uptake in the intermediate group between the two modalities makes it difficult to standardize treatment strategies [46] based on results obtained. A recent systematic review [47] has suggested combined use only in specific circumstances, for instance to characterize indeterminate G2 tumors, to assess disease if there is suspected progression following a period of prolonged stability, or if there is a discrepancy between conventional imaging and clinical/biochemical assessment.

## 3. Peptide Receptor Radiotherapy: From ^111^In to ^177^Lu

Following the success of ^111^In-DTPA-octreotide to diagnose NENs, it was the first radiopharmaceutical to be used as a form of PRRT (peptide receptor radionuclide therapy) soon after for patients with inoperable or metastatic disease. The underlying mechanism for its use was the cytotoxic effect of Auger electrons (low energy electrons with short tissue penetration) ejected from ^111^In following entry into tumor cells following γ emission [48,49]. In an initial case report from 1994, a high cumulative dose of 20 giga-becquerels (GBq) over seven administrations yielded a reduction in volume of a glucagonoma by 20% and transient decline in glucagon and serum GGT levels [48]. The apparent radiation dose to the tumor of 13 gray (Gy) was calculated according to conventional dosimetry, but this was likely an underestimation given the short range of the Auger electrons emitted.

These early promising findings were not entirely validated in further studies; improvements in biochemistry and occasionally of symptoms following therapy were found but tumor regression was not observed as frequently [49,50,51]. Accumulation in bone marrow leading to leukemia and myelodysplastic syndrome was a notable toxic effect as total administered activity levels were titrated to offer maximum clinical efficacy. This was identified in three of six patients who received a total administered activity of 100 GBq in one study, with an estimated bone marrow dose of over 3 Gy [52]. Transient hepatic and renal toxicity were also noted, although these are likely to have related to underlying pathology in affected patients due to tumor replacement of the liver and retroperitoneal fibrosis respectively [52]. The treatment lacked the preferable higher energies of α and β particles, and hence high doses were required to overcome relatively poor soft tissue penetration because of the narrower particle ranges. It was hypothesized at the time that higher energy radionuclides coupled to smaller peptides would lead to more appropriate particle ranges.

Stable conjugation of DOTA-peptides with β-emitting radionuclides such as Yttrium-90 (^90^Y) served as a breakthrough for PRRT. Using DOTA-conjugated somatostatin analogues for imaging enabled selection of patients for these therapies [53] by highlighting SSTR expression of tumors. The physical properties of ^90^Y rendered it very stable, leading to greater investigation of therapeutic radionuclides including ^90^Y-DOTATATE, ^90^Y-DOTA-Lan, and initially, ^90^Y-DOTATOC. The first study to show success of labelling DOTATOC with both ^111^In and ^90^Y was by a team in Basel in 1997 [54], which showed superior biokinetics and a reduced kidney-to-tumor uptake ratio, up to 1.9 times lower, using ^111^In-DOTATOC compared with ^111^In-DTPA-octreotide. It also showed improvement in clinical status of a single patient treated with ^90^Y-DOTATOC. The same study group treated ten more patients with different SSTR positive tumors [55] using ^90^Y-DOTATOC, with three developing partial remission and three demonstrating stable disease. Renal and bone marrow toxicity did not exceed grade 1 of the National Cancer Institute grading criteria and one patient developed grade 2 thrombocytopenia. In anticipation of further therapy trials with ^90^Y-DOTATOC, a further study reviewing biodistribution and dosimetry of ^111^In-DOTATOC showed low absorbed doses in the liver and bone marrow but relatively higher doses to the spleen and kidneys [56]. The adverse consequences of renal uptake were two-fold; namely renal toxicity in itself, and also reduced sensitivity of scintigraphy and therapy due to increased retention of smaller peptides in proximal tubular cells. Following a study in rats, an infusion of positively charged amino acids was found to reduce renal uptake of ^111^In-DTPA-octreotide by up to 40% [57]. Subsequent to this finding, administration of an infusion containing predominantly L-lysine and L-arginine has been used during and after PRRT to reduce renal accumulation of radioactivity.

As the use of ^90^Y-DOTATOC became more established, larger studies exploring the efficacy and toxicity profile were performed. Phase 2 studies in the early 21st century showed response rates of up to 50% over a follow-up period of 6–15 months [58,59]. In one study of 1109 patients published in 2011 which recruited from more than 100 centers, ^90^Y-DOTATOC was administered in cycles of 3.7 GBq/m^2^ each. 12.8% of patients developed grade 3 or 4 transient hematological toxicity and 9.2% developed grade 4 to 5 permanent renal toxicity. Therapeutic responses within the same study were found to be favorable with disease control, either partial response or stable disease, achieved in 39.3% and clinical response in 29.7% over a median follow-up period of 23 months [60]. Studies using ^90^Y-DOTATATE also proved efficacy; for example, a study of 60 patients given 3.7 GBq per cycle on average, showed a median progression-free survival of 17 months and overall survival of 22 months [61]. Whilst the greater energy of ^90^Y showed improved results in metastatic NET, it adversely affected renal function more frequently as doses were up-titrated due to its longer tissue penetration depths of up to 12 mm [14]. In this study, renal toxicity was a delayed manifestation in 11.6% emphasizing close monitoring of kidney function post-therapy [61]. A separate group of 28 patients treated with ^90^Y-DOTATOC had a median decline in creatinine clearance per year of 7.3% [62]. The longer penetration depths also contributed to higher exposures to normal tissues close to tumor sites.

One of the other challenges of using ^90^Y as a radionuclide for PRRT is logistical difficulty in performing dosimetry calculations and tumor imaging, as it has no γ emission for imaging. ^90^Y does produce bremsstrahlung radiation, generated when high energy β^-^ particles slow and lose kinetic energy whilst interacting with adjacent atoms [63]. This slowing process converts kinetic energy into photons. Imaging with bremsstrahlung is limited as there is no dominant energy photopeak of the photons produced, leading to variable counts, inevitable scatter, and low spatial resolution. Whilst these drawbacks do not negate its use in direct dosimetry analysis, in which organ-specific dose estimations can be obtained using planar and SPECT/CT imaging, it is a more labor-intensive and less precise process [64]. Use of a surrogate radionuclide with similar chemistry in lieu such as ^111^In allows for imaging, and a group in Brussels also introduced ^86^Y-DOTATOC PET dosimetry to assess biodistribution [65]. The high energy β emission of ^90^Y of 2.62 MeV, whilst advantageous for targeting tumor cells, can be a potential hazard to operators and requires strict radiation protection measures to avoid excessive finger doses.

Further research continued into potential alternative radionuclides, and ^177^Lu was identified as an encouraging entity given its lower energy β emission of 0.5 MeV and a longer half-life compared with ^90^Y (6.7 days compared to 2.7 days respectively). This offers practical benefits in facilitating transportation over longer distances, improving accessibility. Studies confirmed its effectiveness in therapy, because the absorbed fraction of the lower energy β particle was found to be higher meaning there was a greater absorbed dose to tumor and to micrometastases [66]. A more limited range in tissue compared to ^90^Y also results in less inadvertent irradiation of neighboring structures [67] and lower energies render it less nephrotoxic. Furthermore, ^177^Lu is also a γ-emitter enabling both quantitation and imaging, thus obviating some of the dosimetry considerations needed when using ^90^Y.

In 1998, an international collaborative group known as Specific Peptides for Imaging and Radio Isotope Therapy (S.P.I.R.I.T.) [68] was created to develop radiopharmaceuticals labelled to ligands with specific diagnostic or therapeutic utility, and one of the first peptides devised from this group was ^177^Lu-DOTA, Tyr^3^octreotate or ^177^Lu-DOTATATE, containing a DOTA-peptide, which has a greater affinity for SSTR2 than DOTATOC. After it was shown to be a successful agent at yielding tumor regression and survival in a rat model [69], the first clinical studies using ^177^Lu-DOTATATE started in the Netherlands in 2000. An early comparison of ^177^Lu-DOTATATE and ^111^In-DTPA-octreotide in six patients showed uptake was three-to-fourfold higher for four of five tumors with the former, whilst resulting in no additional dose to the kidneys, spleen, and liver [70]. A preliminary clinical study of 35 patients was subsequently published in 2003, which showed that ^177^Lu-DOTATATE therapy for the treatment of GEP NENs resulted in complete remission in one patient (3%) and partial remission in 12 patients (35%) and no serious side effects during a follow-up period of 3–6 months [71]. These promising findings were substantiated in larger studies which followed, including one of 131 patients in which complete or partial remission was observed in 28%, with minor response or stable disease in a further 54%. Consistent with these findings, the same center undertook toxicity and efficacy analyses in 504 and 310 patients respectively in a study published in 2008 [72]. In this study, patients with SSTR positive disease were treated with a total dose of 27.8–29.6 GBq over a number of treatment cycles, usually four intended cycles of 7.4 GBq each in intervals of 6 to 10 weeks. It was well tolerated overall; WHO grade 3 or 4 hematological toxicity was discovered after only 3.6% of administrations, whilst serious adverse events as a result of the treatment only occurred in five patients (myelodysplastic syndrome in three patients and reversible hepatotoxicity in two patients). 30% of patients had a complete or partial remission and a further 16% had a minor tumor response. Progression-free (33 months) and overall (46 months) survival was also favorable compared to chemotherapy available at the same time period. Quality of life and symptom scales also improved as a consequence of ^177^Lu-DOTATATE therapy in another study of 265 patients with metastasized GEP or bronchial NENs, though a placebo effect may have contributed [73].

Whilst these and numerous other retrospective studies were encouraging, it was not until global manufacturing was established and regulatory approval was negotiated with the European Medicines Agency and FDA that the landmark phase 3 Neuroendocrine Tumors Therapy (NETTER-1) trial came to fruition [74]. This was a prospective international open-label trial, conducted at 41 institutions in patients with progressive SSTR positive midgut NENs. In this trial, 229 patients were randomly assigned to either 7.4 GBq of ^177^Lu-DOTATATE every 8 weeks for four treatment cycles with 30 mg of octreotide long-acting repeatable (LAR) after each treatment followed by octreotide LAR every four weeks; or octreotide LAR alone every four weeks. The primary endpoint of assessing progression-free survival was met at the time of interim analysis, where the ^177^Lu-DOTATATE group showed significantly increased progression-free survival at 20 months compared with the octreotide LAR group (65.2% versus 10.8%). The objective response rate was also significantly greater, at 18% versus 3%. Grade 3 or 4 myelosuppression occurred in fewer than 10% of patients in the ^177^Lu-DOTATATE group and there were no concerns of long-term renal toxicity. Grade 3 and 4 immediate or early adverse reactions found in the ^177^Lu-DOTATATE arm were lymphopenia, deranged liver function, nausea and vomiting, hyperglycemia and hypokalemia, however all were transient and each in fewer than 10%. More common side effects included grade 1 or 2 nausea and vomiting, attributable to the concurrent amino acid infusion with rapid cessation of symptoms following completion, fatigue, abdominal pain and diarrhea.

Supplemental analyses performed after this initial data was published demonstrated statistically significant improvements in quality-of-life indicators including physical functioning, fatigue and pain [75]. In addition, 200 patients entered long-term follow up and could receive further anti-cancer treatment as needed, which showed a clinically meaningful improvement in median overall survival in the ^177^Lu-DOTATATE group of 48 months compared to 36.3 months in the octreotide LAR group. Whilst this was not a statistically significant result, this may have been influenced by a high rate (36%) of cross-over of patients from the octreotide LAR group to the PRRT group [76]. Reassuringly, no new safety concerns were revealed during this long-term follow-up period.

A further retrospective study was published at a similar time point to the interim NETTER-1 publication, which assessed toxicity in 610 patients and efficacy and survival in 443 patients with GEP and bronchial NETs [77]. Findings were concordant with those previously described, with a satisfactory long-term safety profile showing myelodysplastic syndrome in 1.5% of patients and acute leukemia in 0.7%. Grade 3 or 4 hepato- or nephrotoxicity were rare occurrences at 3% and 0.3% respectively and found only to be temporary. The latter was likely due, at least in part, to the co-infusion of L-lysine and L-arginine, which has been found to lower median renal doses by 47% [78]. Outcome data showed median progression-free survival of 29 months and median overall survival of 63 months, and those with liver or bone metastases at baseline had a poorer prognosis.

**Figure 3:**
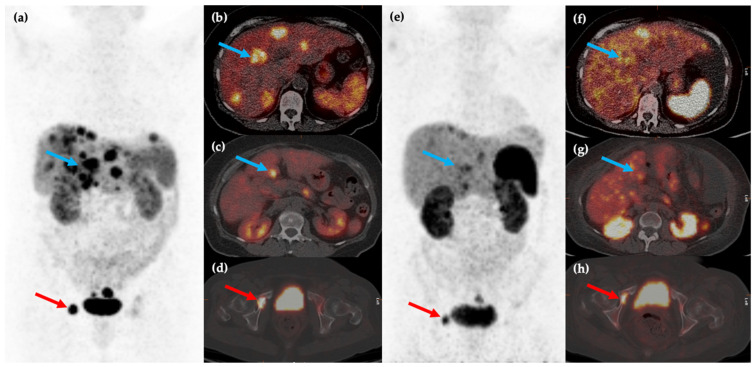
Fifty-year-old female with metastatic G1 NET to liver and bones. ^68^Ga-DOTATATE PET/CT MIP (**a**) and axial (**b**–**d**) images show multiple sites of SSTR positive hepatic (blue arrows) and osseous (red arrows) disease. The patient was treated with ^177^Lu-DOTATATE with favorable partial response on post-therapy ^68^Ga-DOTATATE PET/CT MIP (**e**) and axial (**f**–**h**) images, which show reduction in size and/or number of the previous sites of disease.

These two studies showed that PRRT with ^177^Lu-DOTATATE can be an effective treatment, providing symptomatic benefit whilst being well-tolerated and with low levels of toxicity, although it should be noted that complete response to this treatment remains rare. A meta-analysis of six separate studies with 473 patients concluded that ^177^Lu-DOTATATE is an effective treatment for patients with inoperable or metastatic NENs, with disease response rates ranging between 7% and 43.8% and disease control rates ranging between 73.9% and 100% [79]. The findings of multiple studies led to the eventual approval of ^177^Lu-DOTATATE by the European Commission and the United States Food and Drug Administration (FDA) and this therapy is now utilized for treatment of metastatic or inoperable well-differentiated (G1 or 2) SSTR positive GEP NENs as a second-line treatment (Figure 3).

## 4. Current Therapeutic Considerations with ^177^Lu-DOTATATE

Currently, ^177^Lu-DOTATATE is licensed for use in G1 or G2 NENs. G3 neoplasms can either be well-differentiated (G3 NETs) or poorly differentiated (G3 NECs), and studies in well-differentiated G3 NETs have found PRRT (using both ^177^Lu-DOTATATE and ^90^Y-DOTATOC) to be an effective treatment [80,81,82]. A pooled analysis of three of the larger available studies in patients with G3 GEP NENs showed improved outcomes using PRRT in patients with lower mitotic rates, describing progression-free survival of 11–16 months and overall survival of 22–46 months in those with a Ki-67 21–55%, compared to 4–6 months and 7–9 months in patients with a Ki-67 >55% [83]. Due to heterogeneity of SSTR positivity and biological behavior in tumors with Ki-67 20–55%, some centers advocate additional imaging with ^18^F-FDG PET to provide additional prognostic information and fully characterize all sites of disease, as PRRT may be inappropriate, or at least less successful, if there is a significant burden of SSTR negative and/or FDG positive disease [83]. It has been shown in studies that patients with heterogenous SSTR expression on target lesions had significantly lower time to progression using PRRT compared to those with homogenous expression, of 26 months compared to 54 months [84]. Intuitively, ^18^F-FDG PET may also add value in certain other scenarios, for example when there is rapid progression on anatomical imaging or lesions are seen on anatomical imaging which are not SSTR positive suspicious of tumor heterogeneity [85]. A systematic review and meta-analysis exploring the clinical utility of ^18^F-FDG PET before PRRT showed that disease control rate was higher in patients with a negative ^18^F-FDG PET (91.9%) compared to those with a positive ^18^F-FDG PET (74.2%), making it a useful tool in predicting response and survival outcome [86]. A dual-tracer scoring system called ‘NETPET’ was developed to compare SSTR and FDG positivity, on the basis that more avid lesions on ^18^F-FDG PET compared to SSTR PET is more likely to represent an aggressive phenotype [87]. Further to this, it has been shown that patients with a higher NETPET grade, that is, a higher ratio of FDG to SSTR uptake, are unlikely to derive tumor control from PRRT and should have systemic chemotherapy; whilst those with uptake on both FDG and SSTR imaging are more likely to benefit from combination therapy such as PRRT with chemotherapy [85].

As well as GEP NENs, PRRT has shown potential use in SSTR positive bronchopulmonary NETs, and in selected patients with paragangliomas and phaeochromocytomas, which may be SSTR positive and MIBG negative [88]. Meanwhile, high-grade NECs have higher mitotic rates and tend not to express SSTRs, hence PRRT is not a treatment option. Instead, systemic chemotherapy is the standard of care in this group and also in advanced pancreatic NENs when there has been failure of other therapies [89]. Systemic chemotherapy often comprises platinum-based agents in combination with other agents, commonly cisplatin and etoposide. They can also be used in special circumstances such as bulky disease, rapid symptom or tumor progression, or as a neoadjuvant option if it is felt that there may be a chance of achieving a response sufficient to permit surgery [90].

Alternative treatment options have been introduced more recently in the management of progressive, well-differentiated, metastatic, G1-G3 NETs, including the multiple tyrosine kinase inhibitor (TKI) sunitinib, approved for NETs of pancreatic origin, and mammalian target of rapamycin (mTOR) inhibitor everolimus, used for pulmonary and GEP-NETs. These treatments similarly do not provide a cure but offer potential to stabilize disease and extend progression-free survival after failure of somatostatin analogues. Both have been evaluated in phase 3 studies which have shown positive results compared to placebo. In one study, 171 patients with advanced, well-differentiated pancreatic NENs were randomly assigned in a 1:1 ratio to sunitinib or placebo, and both median progression-free survival (11.4 months versus 5.5 months) and objective response rate (9.3% versus 0%) were higher in the sunitinib group [91]. In another study (entitled RADIANT-3) of 410 patients with advanced low-or intermediate grade pancreatic NENs, who received either everolimus or placebo, median progression-free survival (11 months compared to 4.6 months) and objective response rate (5% versus 2%) were again higher in the everolimus group [92] Similar positive findings were also observed in the subsequent RADIANT-4 study comparing progression-free survival using everolimus and placebo (11 months and 3.9 months) in 302 patients with non-functioning well-differentiated lung or gastrointestinal NENs [93].

A paucity of trial data comparing like with like in G1-G3 NETs impacts on the optimal sequence of these systemic therapies, and available guidelines reflect this relative uncertainty. The European Society of Medical Oncology (ESMO) has stated that because randomized controlled trial data is lacking in pancreatic NENs, molecular targeting agents such as everolimus and sunitinib may be preferred before consideration of PRRT, whilst PRRT can be used earlier in the treatment pathway for management of small intestinal NENs [89]. Practically, however, many centers opt to use PRRT earlier in the treatment algorithm for SSTR positive pancreatic NENs. In addition, the EMSO guidance states that everolimus should be used with caution, if at all, for patients with functioning or advanced NETs. This was after the RADIANT-2 study of 429 patients which compared this therapy plus octreotide LAR to placebo plus octreotide LAR among patients with advanced NETs with carcinoid syndrome, which showed no significant difference in overall survival in the two arms [94]. A recent meta-analysis comparing independent studies of ^177^Lu-DOTATATE and everolimus in treatment of advanced pancreatic NETs showed better objective response rates (47% versus 12%) and disease control rates (81% versus 73%) using ^177^Lu-DOTATATE, though head-to-head comparisons are needed [95]. Sunitinib currently has no role in treatment of gastrointestinal NETs. Liver-directed therapies including debulking surgery, radiofrequency ablation, chemo-or radio-embolization are options in selected patients with hepatic-predominant disease [96] but the correct use of these competing treatments also remains controversial and is often based on patient factors and local expertise [90].

Appropriate patient selection is critical before PRRT is used and multidisciplinary team involvement is mandated. Table 2 outlines the key recommendations pertaining to suitability for PRRT, specifically ^177^Lu-DOTATATE.

In preparing patients and ensuring safety for PRRT, blood tests should be performed for full blood counts, renal function and liver function before and after each treatment cycle to ensure safety of treatment. Dose reduction or discontinuation of treatment may be required if there is derangement in blood tests leading to grade 3 or 4 hematological, renal or hepatic toxicity, or dosing intervals can be increased [97]. On the day of treatment, antiemetics are recommended to combat the common side-effect, and an infusion of L-lysine and L-arginine subsequently given intravenously to reduce renal toxicity which spans the PRRT administration period. Typically, ^177^Lu-DOTATATE is given as 5.5–7.4 GBq over three to five cycles, at an interval of 6–12 weeks between cycles, whilst ^90^Y-DOTATATE or ^90^Y-DOTATOC are given at administered activities of 2.78–4.44 GBq over two to four cycles, also at an interval of 6–12 weeks between cycles [99]. Patients are required to be compliant with radiation protection advice post-treatment and hence need to have appropriate capacity. They must limit close contact with others for 7 days and be particularly cautious with children and pregnant women, adhering to good hygiene to avoid contamination. A risk-benefit analysis is intuitively needed for patients with a history of incontinence [97].

There are special considerations in cases of hormonal crisis (also known as carcinoid crisis). This can arise more commonly in patients with functioning tumors and with poor pharmacological symptom control but is overall considered rare with an occurrence rate of 1% in one study [100]. Sudden massive release of bioactive mediators has been implicated, which cause alterations in fluid dynamics leading to hemodynamic instability, arrhythmia, metabolic acidosis, and alteration in mental status [101,102]. Susceptible patients should be considered for overnight hospitalization and closer clinical review, especially during the first cycle to monitor for these features. Recommended treatments include intravenous boluses of high dose somatostatin analogues to achieve symptom control followed by a continuous infusion, corticosteroids, intravenous fluid resuscitation and correction of electrolyte imbalances. Medications blocking histamine receptors (ranitidine and chlorphenamine) have also been suggested on the assumption that radiation-induced tumor lysis can be contributory [103]. Although somatostatin analogues are generally avoided in the period before PRRT to avoid interference with the treatment, high-risk patients can be considered for pre-treatment and maintenance octreotide [102]. Other prophylactic measures depend on the nature of the functioning syndrome and include pre-hydration, proton-pump inhibitors, anti-emetics, anti-diarrheal medication, and rectification of biochemical abnormalities [104]. Prolonged use of steroid medication should be avoided as studies have shown that they can downregulate SSTR2 receptors [105].

Another factor influencing treatment includes the distribution of disease, particularly relating to liver and bone involvement. A post-hoc analysis of patients in the NETTER-1 study showed that patients even with a high burden of liver disease at baseline did not show increased grade 3 or 4 liver synthetic dysfunction even following ^177^Lu-DOTATATE treatment and showed no significant difference in progression-free survival compared to those with a mild or moderate liver tumor burden [106]. A smaller study assessing safety and efficacy of ^177^Lu-DOTATATE in 11 selected patients with florid bone metastases involving greater than 50% of the axial skeleton showed that although grade 3 or 4 myelotoxicity occurred in four patients (35%), these were temporary and either resolved spontaneously or with supportive measures including transfusion or deferral of therapy for a cycle [107]. If toxicity develops, guidance recommends withholding the next dose of ^177^Lu-DOTATATE until return to baseline followed by resumption of therapy at half of the original dose, 3700 MBq [97]. High tumor burden, especially within the liver, has been linked to an increased risk of carcinoid crisis and additional precautions may be recommended as stated above [103].

## 5. Future Directions for PRRT

Optimization of current protocols including therapy cycles, administered activity, and repeat therapies, and research into novel peptides and radionuclides, are likely to result in better patient outcomes in years to come. Whilst current practice using ^177^Lu-DOTATATE adopts a ‘one size fits all’ fixed dosing regimen spread over four cycles, there is the option to use patient-specific dosimetry to adjust levels of administered activity by measuring lesion and organ doses using several methods. Quantitative three-dimensional modalities such as SPECT/CT depict non-uniform uptake in organs and tumors improving accuracy [108]. Although nephrotoxicity and hemotoxicity are limiting factors when using PRRT, particularly, ^90^Y, the maximum tolerable dose of ^177^Lu-DOTATATE is yet to be defined [109,110] and this provides greater incentive to gather data and harmonies methodologies on patient-specific dosimetry to provide more reliable organ thresholds. The currently held consensus is that individualized kidney and bone marrow dosimetry is likely to contribute to improved outcomes due to impact on tumor doses and wide variation between patients [108]. Indeed, a simulation study in 36 patients who underwent PRRT showed that using a personalized regime would have resulted in a 1.48-fold increase in cumulative maximum tumor absorbed dose whilst maintaining kidney and bone doses at safe levels [111].

Studies have evaluated the use of re-treatment with PRRT as a salvage therapy and shown that those who have previously responded well to ^177^Lu-DOTATATE may again respond well to another course as they progress, although the duration of progression-free survival is lessened [112]. A large study of 181 patients with bronchial and GEP-NETs selected for re-treatment showed similar safety profiles of salvage treatment with another two cycles compared to that of initial PRRT [113], with cumulative doses of up to 60.5 GBq. In the same study, efficacy analyses were performed in 168 patients which showed encouraging progression-free survival periods of over 14 months. Use of supplementary tools, such as a multianalyte assay known as NETest, which has been shown to accurately predict PRRT efficacy, may facilitate improved selection of patients suitable for treatment and re-treatment [114] showing accuracies of 93.7–97.4% as a treatment response biomarker [115].

Timing of treatment requires investigation and PRRT may also be used earlier, or even as a first-line agent. The NETTER-2 trial is underway, a phase 3 trial comparing ^177^Lu-DOTATATE with 30 mg octreotide LAR versus 60 mg octreotide LAR, as a first-line treatment of G2 and G3 advanced GEP NETs [116]. The COMPETE trial is another exciting phase 3 randomized trial comparing ^177^Lu-DOTATOC and everolimus as first-line treatment of advanced GEP NETs of all grades [117]. Similar prospective studies comparing the variety of systemic treatments will be helpful to clarify the optimal treatment pathway.

Neoadjuvant use of PRRT has been described in case reports in patients with pancreatic NENs who were operated on successfully post-treatment [118,119]. A study of post-operative compared outcomes of 23 patients with pancreatic NENs who underwent neoadjuvant PRRT with 23 patients who underwent upfront surgery. There were no differences in intra or postoperative outcomes, but the risk of pancreatic fistula was lower in the PRRT group (0% versus 17%) and progression-free survival in those who achieved an R0 resection was greater in the PRRT group (not reached versus 36 months) [120]. Adjuvant treatment after surgery to prevent tumor spread when micrometastasis or tumor spill was demonstrated at the time of operation has also been found efficacious in animal models [121]. In 94 patients with G1 or two pancreatic NETs with synchronous liver metastases, 31 patients who underwent debulking surgery before PRRT using either ^177^Lu-DOTATATE or ^90^Y-DOTATOC showed significantly improved objective response and progression-free survival compared to 63 non-operated patients who had PRRT only (70 months versus 30 months) [122]. Modifications in administration has been studied and several groups have shown that intra-arterial administration of radiolabeled somatostatin analogues locally led to increased uptake of radioactivity in liver metastasis, thought to result in improved tumor response rates compared to intravenous therapy [123,124].

Whilst ^177^Lu-DOTATATE is favored by many departments over ^90^Y-DOTATOC due to its superior toxicity profile and more less extensive dosimetry, tandem treatment using both ^90^Y and ^177^Lu-based somatostatin analogues can offer synergistic benefits by virtue of their different physical properties (Table 3). This was first shown in animal experiments [125] based on the hypothesis that ^90^Y-labelled somatostatin analogues are more effective for larger tumors, owing to higher energies and deeper tissue penetration resulting in a greater ‘bystander’ effect on adjacent cells, whilst ^177^Lu-labelled somatostatin analogues are more effective for smaller tumors [126]. A cohort study of 486 patients with metastatic NENs showed that tandem treatment was associated with improved overall survival compared to ^90^Y-DOTATOC alone (5.51 versus 3.96 years) with comparable levels of toxicity between the two groups [127]. Combination therapies may be the way forward, yielding better results compared to using either therapy alone [128]. This can facilitate treatments adapted according to the size and distribution of metastatic deposits identified on imaging. However, this is not currently recommended; prospective, randomized studies are needed to confirm improvements in progression-free survival using combination radionuclide therapy before this is clinically implemented [129]. This also needs to be weighed against potential nephrotoxic consequences, which have been shown to be significantly more common both transiently and persistently in patients treated with ^90^Y and combination ^90^Y + ^177^Lu compared with ^177^Lu alone in a study of 807 patients [130].

Use of α-emitters is also an area of active research, as radionuclides such as ^213^Bi and ^225^Ac emit particles with a high energy but with short particle ranges of only 50–100 μm. They may therefore be especially useful for smaller tumors and micrometastases by limiting toxic effects on non-target tissue. Cytotoxic effects do not depend on oxygen concentration; thus, they can be more effective in hypoxic conditions [131,132]. On the contrary, a limitation of the shorter particle range is a less effective ‘crossfire’ effect whereby adjacent tumor cells can also be irradiated; this can typically be beneficial in larger tumors which may be poorly vascularized or heterogenous [133]. The first in-human experience of using ^213^Bi-DOTATOC in seven patients previously treated with a radiolabeled β-emitter showed a positive response to treatment over a two-year follow-up period, with less chemotoxicity [134]. A novel α-emitter in the experimental phase is ^212^Pb-DOTAMTATE, which has had positive clinical implications in animal models [135]. Combinations of α- and β-emitters might aid directed treatment of tumors of different sizes, paralleling many of the perceived advantages of tandem treatment with ^90^Y and ^177^Lu, but production challenges may delay translation into clinical practice. Differences between α- and β-emitters are described in Table 4 [133].

Combining PRRT with other therapies including liver-directed therapies or systemic treatments such as everolimus and sunitinib likewise requires further exploration. For example, sequential treatment using ^90^Y-microsphere selective internal radiation therapy (SIRT) in patients with hepatic progression after PRRT has shown benefit [136]. A phase 1 study in 16 patients evaluating combined everolimus and four cycles of ^177^Lu-DOTATATE showed an overall response rate of 44% with no progression over the treatment duration [137] and a more significant response in four out of five patients with pancreatic NETs. Whether tandem treatment can result in extended improvement in progression-free survival is yet to be seen and can only be answered by high-quality prospective studies. Since the ultimate role of most of the second-and third-line treatments is to halt rather than reverse tumor spread, the counterargument is that using all available treatments too early may leave fewer options in our arsenal at a later stage where it is arguably more necessary. There are concerns regarding use of PRRT after liver-directed therapies due to a greater risk of hepatic radiation toxicity in patients who have had prior radioembolization [138].

Combination cytotoxic chemotherapy with PRRT, known as peptide receptor chemoradionuclide therapy or PRCRT has been investigated with interest and studies have shown therapeutic benefit. These are predominantly from Australia where groups have used PRCRT for over two decades, mostly using concomitant 5-fluorouracil or capecitabine [139,140]. Most recently, a phase 2 trial in 37 patients with G1-3 GEP NETs and both SSTR and FDG positive disease, indicating aggressive tumor behavior, use of ^177^Lu-DOTATATE with capecitabine showed disease stability in 55% and partial response in 30% of patients over a follow-up period of 38 months, and progression-free survival of 31.4 months [141]. A randomized control trial is planned by the same study team. However, there is concern over increased risk of therapy-related myeloid neoplasms [142] and also caution has been recommended in patients with bone metastases due to increase in grade 4 anemia and thrombocytopenia [139]. Synergistic use of more innovative therapies such as the poly (ADP-ribose) polymerase-1 (PARP-1) inhibitor Olaparib has been shown to increase sensitivity of tumor cells to PRRT in vitro [143]. Radiotherapy has been found to increase antigenicity and promote antigen presentation to augment T-cell destruction of tumor cells [144]. In a mouse model, PRRT has been shown to induce an antitumor immune response [145] and employment of immunotherapy alongside PRRT may be another future development. This may be of increased relevance in patients with higher grade tumors in whom expression of programmed death-ligand 1 (PD-L1), a transmembrane protein involved in downregulating the immune response to tumor cells, is higher [146]. Combined PRRT and PD-L1 inhibitors may offer improvements in prognosis for such patients.

SSTR antagonists have shown promise and evidence indicates that they have greater binding capacity to the SSTR and that, although not internalized, they could deliver higher doses of radiation to cells with only reversible minor adverse events. The first safety and efficacy study in 4 patients showed a 1.7–10.6 times higher tumor dose of antagonist ^177^Lu-DOTA-JR11 compared to ^177^Lu-DOTATATE, while tumor-to-kidney and tumor-to-bone marrow doses were also 1.1–7.2 times higher [147]. Feasibility has also been shown in SSTR imaging, with higher tumor-to-background ratios and low liver background uptake compared to currently available ^68^Ga-DOTA-peptides which may be advantageous for detection of metastases [148]. There is further work to be done to determine viability, after a subsequent phase I study in 20 patients with advanced SSTR2 positive NETs treated with radiolabeled SSTR antagonist ^177^Lu-satoreotide tetraxetan. This showed that although response or disease stability were shown in 85%, grade 4 hematological toxicity occurred in 57% of patients after the second cycle [149].

Alternative target receptors could also be targets of future research, after in vitro studies have shown overexpression of peptide receptors other than SSTR on GEP NENs, such as cholecystokinin-2 (CCK2) receptors, glucose-dependent insulinotropic polypeptide (GlP) receptors [150,151]. CCK2 receptors are also overexpressed in other NENs including medullary thyroid cancer and insulinoma [152]. If successful in-vivo, these would provide alternative options for patients with NENs with low or absent SSTR expression or could be used in combined or sequential administrations for tumors expressing a variety of receptors. Other novel compounds being studies include miniaturized drug conjugates such as PEN-221, a SSTR2-binding somatostatin analogue linked to the microtubule inhibitor mertansine (DM1) [153]. After showing preliminary efficacy in a phase 1/2a study [154], a phase 2 study in 32 patients with advanced midgut NETs demonstrated that it was well-tolerated with adverse events of grade 3 or greater in only 10%, with a clinical benefit rate of 88.5% and median progression-free survival of 9 months [155]. A randomized trial is now in development.

PRRT has shown disease control in metastatic and inoperable NENs beyond those of gastro-entero-pancreatic origin, such as SSTR positive paragangliomas and phaeochromocytomas [156,157] and a paper which compared reports of efficacy of PRRT in these patients showed either partial response or stable disease in at least 67% of patients, although studies to date have been limited by small sample sizes [158]. The same paper reported findings of the use of combined PRRT and the chemotherapeutic agent capecitabine in 25 patients with malignant paraganglioma. It proved efficacy with objective response in 28% and symptomatic response in 43%, but there was no great advantage of concomitant therapy compared to published PRRT monotherapy outcomes. This remains an area of future research as study numbers to the present time have been low. A phase 2 trial is underway evaluating the use of ^177^Lu-DOTATATE in inoperable phaeochromocytomas and paragangliomas [159] and is due to be completed in 2024. There is capacity to extend the role of PRRT even further and early studies in patients with refractory SSTR positive metastatic neuroblastoma showed potential using a combination of ^177^Lu-DOTATATE, ^90^Y-DOTATOC and ^111^In- DOTATATE [160,161,162]. Unfortunately, a more recent phase 2 trial from the United Kingdom in 14 patients with relapsed and refractory neuroblastoma did not report similar levels of objective response [163]. In spite of this, there is continued interest in PRRT for this indication, and a novel theranostic pairing of ^64^Cu-SARTATE for imaging and ^67^Cu-SARTATE for treatment is being explored after feasibility was shown in animal models [164,165].

Results of a Dutch study proving efficacy and safety of ^177^Lu-DOTATATE in metastatic grade 1 and 2 bronchial NETs [77] were encouraging, although this is currently an off-label indication. A later study of 25 patients treated with either ^177^Lu-DOTATATE or ^90^Y-DOTATATE showed a median progression-free survival of 17 months, which is comparable if not favorable to other systemic therapies [166] but larger trial data are required [167] and a phase 2 trial comparing it to everolimus in bronchial NETs is due to recruit [168]. Combination treatments used in refractory small cell lung cancer, a high-grade NEN, are also being investigated. A pre-clinical study reported that combination treatment with ^177^Lu-DOTATATE and carboplatin/etoposide chemotherapy in mouse models with SSTR expressing small cell lung cancer was more effective than either treatment alone [169] and this is to be translated to human studies. A phase 1 study conducted in patients with relapsed or refractory extensive-stage SSTR positive small cell lung cancer showed that a combination of ^177^Lu-DOTATATE and the anti-PD-1 inhibitor nivolumab was well-tolerated and showed signs of antitumor activity [170]. Additional studies are warranted to assess effectiveness. A systematic review of 41 papers evaluating the use of PRRT in a subgroup of patients with SSTR positive radioiodine-refractory differentiated thyroid cancer and metastatic medullary thyroid cancer showed biochemical responses of 25.3–37.2% and objective response in 10.5–10.6%, with few adverse events identified [171]. It should be noted that individual study sizes were variable and the types of PRRT used, and patient populations were heterogenous, so the review concluded that multi-center randomized controlled trials are recommended to validate against other currently available treatments. At present, ESMO and the American Thyroid Association agree that current experience of PRRT in medullary thyroid carcinoma is limited and do not endorse use, but conceivable benefit when other modes of management such as tyrosine kinase inhibitors are contraindicated is recognized [172].

## 6. Conclusions

PRRT has been found to be a highly effective and well-tolerated treatment for metastatic, unresectable SSTR positive neuroendocrine neoplasms. Though it is currently considered a second-line treatment and there are several other options for patients with disseminated disease, further prospective trial evidence is required to ascertain whether more widespread use earlier in the management pathway is conceivable and to determine whether competing treatment options currently available may have complementary roles. There may also be an evolving role of tandem PRRT therapies, tailored to the distribution of disease, in the quest for truly personalized treatment.

## Figures and Tables

**Table 1:** WHO classification of Gastroenteropancreatic NEN.

WHO 2019 Gastrointestinal NEN Classification	WHO 2017 Pancreatic NEN Classification	WHO 2010 Gastroenteropancreatic NEN Classification
Well-differentiated NETs: • NET G1 • NET G2 • NET G3	Well-differentiated NETs: • NET G1 • NET G2 • NET G3	Well-differentiated NETs: • NET G1 • NET G2
Poorly differentiated NECs: • NEC (large cell or small cell NEC)	Poorly differentiated NECs: • NEC G3 (large cell or small cell NEC)	Poorly differentiated NECs: • NEC G3 (large cell or small cell NEC)

G1/Low grade = Mitotic rate < 2 per 2 mm^2^ or Ki-67 < 3%, G2/Intermediate grade = Mitotic rate 2–20 per 2 mm^2^ or Ki-67 3–20%, G3/High grade = Mitotic rate >20 per 2 mm^2^ or Ki-67 > 20%. MiNEN = Mixed neuroendocrine-non-neuroendocrine neoplasm, MANEC = Mixed adenoneuro-endocrine carcinoma.

**Table 2:** Patient selection recommendations for ^177^Lu-DOTATATE.

**Tumor-Related Factors**	
**Imaging**	SSTR positive, metastatic or inoperable NET with disease progression
	Lesion uptake should exceed background hepatic activity
**Histology**	G1/2 NET ideally * Ki-67 ≤20% ideally *
**Patient-Related Factors**	
**Clinical**	Increasing symptoms or disease progression
	ECOG performance status 0–2 or Karnofsy/Lansky performance status above 60% New York Heart Association grade <III Able to comply with radiation protection advice Discontinuation of long-acting ‘cold’ somatostatin analogues for 4–6 weeks Functioning syndromes—consider inpatient treatment
**Renal function**	Creatinine clearance >40 mL/min
**Liver function**	Bilirubin <3 x upper limit of normal ^#^
	Albumin >30 g/L ^#^
**Full blood count**	Hb >8 g/dL WCC >3000/mm ^3^ Platelets >75,000/mm ^3^

* May be used in well-differentiated G3 and Ki-67 20–55% as described, depending on center. ^#^ These are recommended indications by the manufacturers [97] and Society of Nuclear Medicine and Medical Imaging (SNMMI) [98] but are not outlined in the European Association of Nuclear Medicine (EANM) practical guidance on PRRT [99].

**Table 3:** Physical properties of ^90^Y and ^177^Lu.

	^90^Y (Yttrium-90)	^177^Lu (Lutetium-177)
Emission spectrum	β^−^	β^−^ and γ-emitter
Physical half-life (days)	2.7	6.7
Maximum beta energy (MeV)	2.28	0.50
Particle penetration (mm)	11.3	1.8
Imaging ability	Bremsstrahlung	γ emission

**Table 4:** Physical and biological differences between α and β particles.

	α Particles	β Particles
Particle type	^4^He nucleus	Energetic electron
Particle energy	5–9 MeV	50–2300 keV
Particle path length	50–100 μm	0.05–12 mm
Linear energy transfer	~80 keV/μm	~0.2 keV/μm
Oxygenation	Effective in hypoxic tumors	Less effective in hypoxic tumors
Bystander effect	Yes	Yes
Tumor crossfire	Low	Yes
